# Tumor-related epilepsy in high-grade glioma: a large series survival analysis

**DOI:** 10.1007/s11060-024-04787-z

**Published:** 2024-08-05

**Authors:** Ryan G. Rilinger, Lydia Guo, Akshay Sharma, Josephine Volovetz, Nicolas R. Thompson, Matthew Grabowski, Mina Lobbous, Andrew Dhawan

**Affiliations:** 1 Cleveland Clinic Lerner College of Medicine, Case Western Reserve University, Cleveland, USA; 2 Department of Neurosurgery, Cleveland Clinic Foundation, Cleveland, USA; 3 Lerner Research Institute Quantitative Health Sciences Department, Cleveland, USA; 4 Neurological Institute Center for Outcomes Research & Evaluation, Cleveland, USA; 5 Department of Neuro-Oncology, Cleveland Clinic Foundation, Cleveland, USA; 6 Rose Ella Burkhardt Brain Tumor and Neuro-Oncology Center, 9500 Euclid Avenue, Cleveland, OH 44195 USA

**Keywords:** Epilepsy, High-grade glioma, Glioblastoma, Prognosis, Seizure

## Abstract

**Purpose:**

Seizures are a common clinical occurrence in high-grade glioma (HGG). While many studies have explored seizure incidence and prevalence in HGG, limited studies have examined the prognostic effect of seizures occurring in the post-diagnosis setting. This study aims to assess the impact of seizure presentation on HGG survival outcomes.

**Methods:**

Single-center retrospective review identified 950 patients with histologically-confirmed high-grade glioma. Seizure presentation was determined by clinical history and classified as early onset (occurring within 30 days of HGG presentation) or late onset (first seizure occurring after beginning HGG treatment). The primary outcome, hazard ratios for overall survival and progression-free survival, was assessed with multivariable Cox proportional-hazards models. *IDH1* mutation status (assessed through immunohistochemistry) was only consistently available beginning in 2015; subgroup analyses were performed in the subset of patients with known *IDH1* status.

**Results:**

Epileptic activity before (HR = 0.81, 95% CI = 0.68–0.96, *P* = 0.017) or after (HR = 0.74, 95% CI = 0.60–0.91, *P* = 0.005) HGG diagnosis associated with improved overall survival. Additionally, late seizure onset significantly associated with lower odds of achieving partial (OR = 0.25, 95% CI = 0.12–0.53, P = < 0.001) or complete (OR = 0.30, 95% CI = 0.18–0.50, *P* < 0.001) seizure control than patients with early seizure onset.

**Conclusions:**

Clinical seizures both at the time of diagnosis and later during the HGG treatment course are associated with improved overall survival. This association potentially persists for both *IDH1*-wildtype and *IDH1-*mutant patients, but further study is required.

**Supplementary Information:**

The online version contains supplementary material available at 10.1007/s11060-024-04787-z.

## Introduction

High-grade gliomas (HGG), a group of infiltrative neoplasms including Grade IV *IDH*-wildtype astrocytoma (formerly glioblastoma multiforme; GBM), currently affect between 0.59 and 5 individuals per 100,000 population per year [1]. In 2021, the World Health Organization (WHO) amended its definition of GBM to follow genetic guidelines in addition to histological guidelines, requiring a diagnosis of GBM to lack isocitrate-dehydrogenase 1 and 2 mutations (*IDH*-wildtype); IDH-mutant grade IV gliomas are now termed *IDH*-mutant astrocytomas [2].

Seizures are a common clinical occurrence throughout the disease course in HGG, estimated to affect 30–60% of patients with HGG [3, 4]. While many studies have explored the impact of surgical and medical HGG treatment on seizure incidence and prevalence, limited studies have assessed the prognostic effect of seizures occurring in the post-treatment setting [5, 6].

Recently, a positive seizure history at initial HGG presentation has been shown to associate with improved overall survival: a 2017 meta-analysis of 1,836 GBM patients found reduced mortality with positive seizure history (HR = 0.71, P = < 0.00001); a 2018 meta-analysis of 2,088 patients showed increased mortality with negative seizure history (HR = 1.73, P = < 0.001) [7, 8]. Current theories for the underlying mechanism behind a possible protective effect of seizures in HGG include both cellular theories (such as an association with *IDH1* mutation) and clinical theories (such as early detection of HGG through seizure work-up) [7].

In this study, we aimed to retrospectively assess the effects of tumor-related epilepsy in patients with HGG on overall and progression-free survival. Further, we sought to better delineate the temporal relationship between seizure onset and HGG survival to understand whether early or late seizures carry distinct prognoses in tumor-related epilepsy. Given the substantial recent history of including *IDH*-mutant disease in the umbrella of GBM as well as the uncertainty regarding *IDH1* status’s impact, our paper additionally incorporates both *IDH*-wildtype GBM and IDH-mutant grade IV astrocytomas.

## Materials and methods

### Participants

We retrospectively reviewed all patients diagnosed with HGG (defined as WHO Grade IV glioma), histologically confirmed by board-certified neuropathologists, at the Cleveland Clinic between 1999 and 2022. For seizure control analyses, only patients with clinically diagnosed epileptic activity (defined based on neurologist-obtained history or direct observation during hospitalization) were included. Electroencephalogram (EEG) data was not included as it was not collected as part of the standard of care for all patients [3].

### Data collection

Electronic medical records were queried for relevant clinical factors including: age, sex, extent of surgical resection (defined as gross total resection versus all other surgical procedures), laterality, primary tumor location (defined as frontal lobe, parietal lobe, temporal lobe, occipital lobe, or other location), Karnofsky Performance Score (KPS) at diagnosis, radiation therapy, chemotherapy (cytotoxic), chemotherapy (biological target), *IDH1* mutation status, survival duration (progression-free (PFS) and overall (OS)), and presence of epileptic activity. Epileptic activity was classified into three groups: None (no history of epileptic activity), Early Seizure (defined as first seizure occurring within 30 days prior to HGG diagnosis), and Late Seizure (defined as first seizure occurring after HGG diagnosis). “Diagnosis” timepoint was defined as the date of first surgery which provided the tissue sample for histologic confirmation of disease. PFS duration was defined as the time from diagnosis until first progression or recurrence of disease as measured by the Response Assessment in Neuro-Oncology (RANO) criteria [9]; OS was defined as the time from diagnosis until death. Seizure control was classified into three groups: None (never achieved six consecutive seizure-free months), Partial (achieved at least six consecutive seizure-free months, but later relapsed), and Complete (achieved permanent seizure control of at least six consecutive months). Consistent immunohistochemistry for *IDH1* mutations was not available until 2015; *IDH1* mutation status prior to 2015 was sporadic.

### Statistical analysis

Descriptive statistics summarized the patient sample, overall and stratified by seizure status (None, Early Seizure, Late Seizure). Mean with standard deviation or median with interquartile range was used for continuous variables and frequency with percentage was used for categorical variables. Group comparisons were made using one-way analysis of variance (ANOVA) or Kruskal-Wallis test for continuous variables and chi-square or Fisher’s exact test for categorical variables.

Kaplan-Meier curves were constructed for the overall sample to estimate survival and progression-free survival. To determine if timing and severity of initial seizure presentation, as well as seizure control, were associated with survival and progression-free survival, we fit multivariable Cox proportional hazards models. Surviving patients were censored at their date of last follow-up. Separate models were fit for each outcome (survival and progression-free survival) and for each independent variable of interest (seizure presence, timing of initial seizure [None, Early Seizure, Late Seizure], severity of epileptic disease initial seizure presentation [0, 1, 2 + seizures/month], and seizure control [None, Partial, Complete]). In all models, we adjusted for the following covariates: age, sex, gross total resection (vs. subtotal resection or biopsy), laterality (bilateral vs. right/left), frontal lobe, parietal lobe, temporal lobe, occipital lobe, other location, KPS at diagnosis, radiation therapy, chemotherapy (cytotoxic), and chemotherapy (biological target). We used variance inflation factors (VIFs) to assess for multicollinearity with VIF > 5 indicating multicollinearity.

In order to reduce a potential persistence bias for seizure occurring after diagnosis in patients with long survival duration, we performed an analysis restricting the Late Seizure group to those patients with their first seizure within the 14 months following diagnosis. 14 months was chosen as a cutoff to represent the median survival time in IDH-wildtype HGG, the most common type of HGG [1].

We further examined these models in the subset with known *IDH1* wildtype status. Subgroup analysis focusing on seizure control was performed on those patients who had seizures. Seizure control was defined as a categorical variable with three groups: None (never achieved six consecutive seizure-free months), Partial (achieved at least six consecutive seizure-free months, but later relapsed prior to death), and Complete (achieved permanent seizure control of at least six consecutive months). Six months of seizure absence was chosen as the definition for seizure remission. Six months is a commonly-selected window in prior studies of epileptic remission, as well as the most common window required to reattain driving eligibility across US states [10–12].

To assess whether seizure timing (Early Seizure vs. Late Seizure) and initial seizure frequency (0, 1, 2 + seizures) were associated with seizure control, we fit two multivariable multinomial logistic regression models where seizure control was the dependent variable.

## Results

**Fig. 1:**
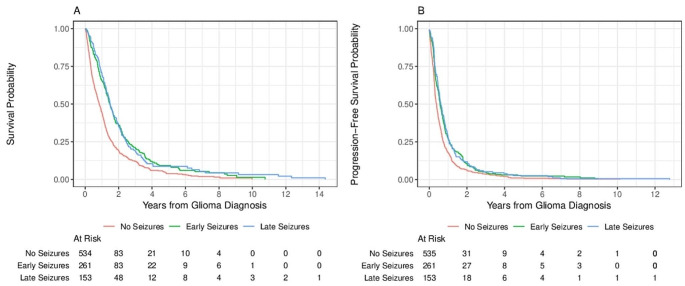
Kaplan-Meier curves for overall (**A**) and progression-free (**B**) survival since glioma diagnosis. Median overall survival time was 13.4 months (95% CI = 12.4–14.3 months). Median progression-free survival was 5.5 months (95% CI = 5.1–6.1 months)

950 patients with a histologically confirmed diagnosis of HGG treated at the Cleveland Clinic Foundation from 1999 to 2022 meeting inclusion criteria were identified. 414 patients (43.6%) had seizures; of these, 261 (63.0%) had their initial seizure before glioma diagnosis, and 153 (37.0%) had their initial seizure following initiation of glioma treatment. Table 1 shows descriptive statistics of patient and clinical characteristics, stratified by seizure status; patients with no history of seizures were significantly older (P = < 0.001) and less likely to have received chemotherapy (P = < 0.001) than patients with Early or Late seizures, but no significant differences between groups were observed for sex (*P* = 0.444). Additional demographic and clinical characteristics are provided in Supplementary Table 1. Further, Supplementary Table 2 classifies all included anticancer therapies as cytotoxic chemotherapy, targeted chemotherapy, or immunotherapy. Figure 1 shows overall survival and progression-free survival for the entire sample. Median overall survival time was 13.4 months (95% CI = 12.4–14.3 months). Median progression-free survival was 5.5 months (95% CI = 5.1–6.1 months).

**Table 1:** Patient and clinical characteristics, stratified by seizure status. “Statistic” is “No. (%)” unless noted otherwise

	All Patients		No Seizures		Early Seizures (pre-HGG diagnosis		Late Seizures (post-HGG diagnosis)		*P*-value
	*N*	Statistic	*N*	Statistic	*N*	Statistic	*N*	Statistic	
**Age**,** mean (SD)**	949	61.0 (13.7)	535	63.7 (13.5)	261	57.6 (13.2)	153	57.2 (13.4)	< 0.001
**Sex**									
Female	950	352 (37.1)	536	208 (38.8%)	261	91 (34.9%)	153	53 (34.6%)	0.444
Male		598 (62.9%)		328 (61.2)		170 (65.1%)		100 (65.4%)	
**Race**									
American Indian/Alaska Native	948	1 (0.1%)	535	0 (0.0%)	260	1 (0.4%)	153	0 (0.0%)	0.727
Asian		6 (0.6%)		3 (0.6%)		2 (0.8%)		1 (0.7%)	
Black		33 (3.5%)		19 (3.6%)		10 (3.8%)		4 (2.6%)	
White		883 (93.1%)		501 (93.6%)		237 (91.2%)		145 (94.8%)	
Other		25 (2.6%)		12 (2.2%)		10 (3.8%)		3 (2.0%)	
**Surgical Intervention**									
Resection	950	648 (68.2%)	536	343 (64.0%)	261	192 (73.6%)	153	113 (73.9%)	0.012
Biopsy + Laser interstitial thermal therapy		33 (3.5%)		17 (3.2%)		9 (3.4%)		7 (4.6%)	
Biopsy Only		269 (28.3%)		176 (32.8%)		60 (23.0%)		33 (21.6%)	
**Resection Type**									
Sub-total Resection	637	192 (30.1%)	336	104 (31.0%)	190	51 (26.8%)	111	37 (33.3%)	0.215
Near-total Resection		151 (23.7%)		87 (25.9%)		38 (20.0%)		26 (23.4%)	
Gross-total Resection		294 (46.2%)		145 (43.2%)		101 (53.2%)		48 (43.2%)	
**Radiation**	944	813 (86.1%)	532	425 (79.9%)	259	242 (93.4%)	153	146 (95.4%)	< 0.001
**Chemotherapy (Cytotoxic)**	945	767 (81.2%)	532	394 (74.1%)	260	237 (91.2%)	153	136 (88.9%)	< 0.001
**Chemotherapy (Biologic Target)**	942	401 (42.6%)	531	169 (31.8%)	259	143 (55.2%)	152	89 (58.6%)	< 0.001
**Follow-up Time (months)**,** median (IQR)**	948	9.1 (2.5, 18.6)	534	5.2 (1. 5, 13.9)	261	13.8 (4.9, 24.7)	153	13.1 (6.4, 22.8)	< 0.001

### The impact of seizures on overall and progression-free survival

In the full patient cohort (*N* = 950), seizure timing was associated with prolonged overall survival but not with prolonged progression-free survival (Table 2). Compared to patients who did not have any seizures, patients with seizures had lower risk of death whether they experienced seizures before (HR = 0.81, 95% CI = 0.68–0.96, *P* = 0.017) or after (HR = 0.74, 95% CI = 0.60–0.91, *P* = 0.005) glioma diagnosis. Initial seizure frequency was not associated with overall survival (omnibus p-value = 0.968) or progression-free survival (omnibus p-value = 0.548).

Of the 153 patients who had their first seizure post-glioma diagnosis, 120 (78.4%) had their first seizure within 14 months after glioma diagnosis. When restricting the Late Seizure group to these 120 patients, patients in the Early Seizure group still had lower risk of death than patients who never had seizures (HR = 0.80, 95% CI = 0.67–0.95, *P* = 0.011); however, the Late Seizure group did not have significantly different risk of death than the no-seizure group (HR = 0.89, 95% CI = 0.72–1.12, *P* = 0.324) (Table 2).

Patients who had any seizures displayed significantly reduced risk of death compared to those who never had seizures (Table 2). This association was seen when analyzing all patients (HR = 0.78, 95% CI = 0.67–0.91, *P* = 0.002) and when excluding patients whose post-glioma seizure diagnosis was greater than 14 months from diagnosis (HR = 0.83, 95% CI = 0.71–0.97, *P* = 0.017). Further, patients with seizures had significantly lower risk of progression when considering all patients (HR = 0.84, 95% CI = 0.73–0.97, *P* = 0.020) but not when excluding patients with post-glioma seizures beginning more than 14 months following glioma diagnosis (HR = 0.90, 95% CI = 0.78–1.04, *P* = 0.166) (Table 2). A separate analysis was run including only patients who received anticancer therapy (*n* = 829), yielding similar findings (Supplementary Table 3).

We next examined patients with known *IDH1*-wildtypestatus (*N* = 563) [13, 14]. Among these patients,, neither seizure timing (omnibus p-value = 0.204 for OS; 0.384 for PFS) nor initial seizure severity (omnibus p-value = 0.431 for OS; 0.320 for PFS) displayed significant associations with overall survival or progression-free survival (Table 2). When classifying patient seizure status as a binary variable (“any timing of seizures” vs. “no seizures”) seizure presence did not display significant associations with overall survival (HR = 0.83, 95% CI = 0.68, 1.02, *P* = 0.075) or progression-free survival (HR = 0.91, 95% CI = 0.75–1.10, *P* = 0.332) (Table 2). In all models, there was no evidence of multicollinearity (all VIFs < 3).

**Table 2:** Multivariable Cox proportional hazard models for overall survival and progression-free survival. Separate models were fit for each outcome and independent variable: seizure timing (early and late seizure vs. no seizures), frequency (number of seizures at initial HGG presentation), and control (partial or complete control vs. no control)

		Hazard Ratio (95% CI)	*P*-value	Omnibus *P*-value^b^
Overall Survival Models (All patients; *N* = 950)				
Timing (vs. No seizure)	Early seizure	0.81 (0.68, 0.96)	0.017	0.005
	Late seizure	0.74 (0.60, 0.91)	0.005	
	Any seizure	0.78 (0.67, 0.91)	0.002	NA
Restricted Timing (vs. No seizure)^a^	Early seizure	0.80 (0.67, 0.95)	0.011	0.037
	Late seizure	0.89 (0.72, 1.12)	0.324	
	Any seizure	0.83 (0.71, 0.97)	0.017	NA
Frequency (vs. 0)	1	1.02 (0.80, 1.31)	0.867	0.968
	2+	1.04 (0.77, 1.40)	0.807	
Seizure control (vs. No control)	Partial control	0.30 (0.21, 0.43)	< 0.001	< 0.001
	Complete control	0.43 (0.33, 0.55)	< 0.001	
**Progression-Free Survival Models** **(All patients**, ***N*** = **950)**				
Timing (vs. No seizure)	Early seizure	0.87 (0.74, 1.02)	0.082	0.055
	Late seizure	0.81 (0.66, 0.98)	0.033	
	Any seizure	0.84 (0.73, 0.97)	0.020	NA
Restricted Timing (vs. No seizure)^a^	Early seizure	0.87 (0.74, 1.02)	0.088	0.213
	Late seizure	0.98 (0.80, 1.22)	0.882	
	Any seizure	0.90 (0.78, 1.04)	0.166	NA
Frequency (vs. 0)	1	0.94 (0.74, 1.20)	0.612	0.548
	2+	1.10 (0.82, 1.47)	0.525	
Seizure control (vs. No control)	Partial control	0.45 (0.32, 0.62)	< 0.001	< 0.001
	Complete control	0.54 (0.43, 0.69)	< 0.001	
**Overall Survival Models** **(*IDH1*wildtype**, ***N*** = **563)**				
Timing (vs. No seizure)	Early seizure	0.83 (0.65, 1.06)	0.129	0.204
	Late seizure	0.83 (0.64, 1.08)	0.169	
	Any seizure	0.83 (0.68, 1.02)	0.075	NA
Restricted Timing (vs. No seizure)^a^	Early seizure	0.83 (0.65, 1.05)	0.116	0.275
	Late seizure	0.90 (0.68, 1.19)	0.463	
	Any seizure	0.85 (0.69, 1.05)	0.133	NA
Frequency (vs. 0)	1	0.98 (0.70, 1.38)	0.919	0.431
	2+	1.27 (0.84, 1.92)	0.259	
		**Hazard Ratio (95% CI)**,** continued**	**P-value**,** continued**	**Omnibus P-value**,** continued**
**Progression-Free Survival Models** **(*IDH1*wildtype**, *N* = **563)**				
Timing (vs. No seizure)	Early seizure	0.97 (0.77, 1.21)	0.772	0.384
	Late seizure	0.84 (0.66, 1.08)	0.174	
	Any seizure	0.91 (0.75, 1.10)	0.332	NA
Restricted Timing (vs. No seizure)^a^	Early seizure	0.97 (0.77, 1.21)	0.788	0.888
	Late seizure	0.94 (0.72, 1.22)	0.641	
	Any seizure	0.96 (0.79, 1.16)	0.663	NA
Frequency (vs. 0)	1	1.09 (0.79, 1.50)	0.601	0.320
	2+	1.42 (0.96, 2.11)	0.080	

^a^ “Restricted Timing” = model including only Late Seizure patients with seizure onset within 14 months of HGG diagnosis

^b^ Omnibus test is not applicable for the Any seizure vs. No seizure models

### Epileptic presentation and severity association with glioma-related seizure control

Both seizure timing and frequency were independently associated with likelihood of achieving seizure control (Table 3). Compared to patients in the Early Seizure group, patients in the Late Seizure group were less likely to achieve partial (OR = 0.25, 95% CI = 0.12–0.53, *P* < 0.001) or complete (OR = 0.30, 95% CI = 0.18–0.50, *P* < 0.001) seizure control. Patients with one seizure in the 30 days before their HGG diagnosis had greater odds of achieving partial (OR = 2.88, 95% CI = 1.28–6.49, *P* = 0.011) and complete (OR = 3.04, 95% CI = 1.75–5.30, *P* < 0.001) seizure control compared to patients with no seizures at presentation. For patients with more than one seizure in the 30 days before their diagnosis, odds of achieving partial seizure control (OR = 4.44, 95% CI = 1.81–10.89, *P* = 0.001) and complete seizure control (OR = 2.65, 95% CI = 1.36–5.16, *P* = 0.004) were also higher than those without seizures at presentation.

**Table 3:** Impacts of seizure timing and initial seizure frequency on likelihood of achieving seizure control (partial or complete). Separate models were fit for each independent variable (seizure timing and seizure frequency)

		Partial Control		Complete Control	
All patients (*N* = 950)		Odds Ratio vs. No Control (95% CI)	*P*-value	Odds Ratio vs. No Control (95% CI)	*P*-value
Seizure timing	Late vs. Early	0.25 (0.12, 0.53)	< 0.001	0.30 (0.18, 0.50)	< 0.001
Seizure frequency	1 vs. 0	2.88 (1.28, 6.49)	0.011	3.04 (1.75, 5.30)	< 0.001
	2 + vs. 0	4.44 (1.81, 10.89)	0.001	2.65 (1.36, 5.16)	0.004
**IDH1-wildtype patients (***N* = **577)**		**Odds Ratio vs. No Control (95% CI)**	**P-value**	**Odds Ratio vs. No Control (95% CI)**	**P-value**
Seizure timing	Late vs. Early	0.16 (0.06, 0.45)	< 0.001	0.23 (0.12, 0.46)	< 0.001
Seizure frequency	1 vs. 0	4.25 (1.37, 13.14)	0.012	5.94 (2.73, 12.92)	< 0.001
	2 + vs. 0	6.50 (1.87, 22.59)	0.003	3.94 (1.52, 10.20)	0.005

Compared to patients who did not achieve seizure control, risk of progression was lower in patients with partial (HR = 0.45, 95% CI = 0.32–0.62, P = < 0.001) or complete (HR = 0.54, 95% CI = 0.43–0.69, P = < 0.001) seizure control. Risk of death was also lower in patients with partial (HR = 0.30, 95% CI = 0.21–0.43, P = < 0.001) or complete (HR = 0.43, 95% CI = 0.33–0.55, P = < 0.001) seizure control compared to those who did not achieve any seizure control.

The *IDH1* wildtype subanalysis demonstrated a significant association of seizure timing with likelihood of seizure control: the Late Seizure group was less likely than the Early Seizure group to achieve partial (OR = 0.16, 95% CI = 0.06–0.45, P = < 0.001) or complete (OR = 0.23, 95% CI = 0.12–0.46, P = < 0.001) seizure control (Table 3). For patients with a pathogenic variant in *IDH1*, timing of first seizure and seizure frequency did not display significant associations with odds of attaining partial or complete seizure control (Table 4).

**Table 4:** Frequency and percentage of seizure control by seizure timing and by seizure frequency in patients with *IDH1* mutation

	No Control	Partial Control	Complete Control	*P*-value
Seizure Timing				
Early Seizure	4/19 (21%)	3/19 (16%)	12/19 (63%)	0.568
Late Seizure	2/4 (50%)	0/4 (0%)	2/4 (50%)	
**Seizure Frequency**				
0	4/10 (40%)	0/10 (0%)	6/10 (60%)	0.225
1	2/10 (20%)	3/10 (30%)	5/10 (50%)	
2+	0/3 (0%)	0/3 (0%)	3/3 (100%)	

## Discussion

We present a retrospective analysis of 950 patients with HGG and recorded seizure status, demonstrating an association with improved survival for both patients with seizure activity at baseline and patients who first developed seizure activity after their HGG diagnosis. Our approach allowed us to explore whether epilepsy itself associates with prolonged survival or whether the effect is confounded by a lead-time bias of earlier HGG diagnosis (extending survival only in patients who had epileptic activity prior to HGG diagnosis).

Consistent with prior studies, seizure presence at disease presentation was associated with improved overall survival, but not progression-free survival, within our full 950-patient cohort [8, 15]. A 2018 meta-analysis including 368 patients found an increase in progression-free survival among patients with epilepsy at initial glioma presentation; however, this sample included a mix of low-grade (LGG) and high-grade gliomas [7]. The incidence of seizure activity in LGG is higher than in HGG; our decision to focus exclusively on HGG may explain our different findings [6]. Additionally, PFS is influenced by inter-radiologist differences in scan interpretation as well as follow-up cadence; this subjectivity may contribute to differences in PFS conclusions between studies [16]. We believe that OS is a much more objective measure of disease-related longevity. Alternatively, it is possible that seizures associate with increased survival via cancer-independent mechanisms (e.g. more frequent follow-up with medical providers, potential unknown benefits of ASM use), thereby linking to OS but not affecting PFS.

A 2024 retrospective analysis by Pallud et al. of *IDH1*-wildtype GBM yielded similar findings regarding the prognostic impact of seizure presence at disease presentation; we add to this analysis by also reporting a previously undescribed significant increase in both overall and progression-free survival among patients whose first seizure occurred after histologically-confirmed diagnosis of HGG (our “Late Seizure” group) [15]. To our knowledge, our analysis is one of the first and largest cohorts examining the prognostic value of seizures presenting after HGG diagnosis. By finding a significant association between seizures and survival in patients who experienced their first seizure after HGG diagnosis (even after accounting for exposure to anticancer therapy), our data suggests that the positive prognostic impact of epilepsy goes beyond the explanation of lead time bias proposed in prior studies [17, 18].

Neuronal hyperexcitability has recently been implicated as a potential driver for gliomagenesis and tumor progression [19]. Peritumoral glutamatergic neurons can synapse directly on tumor cells; hyperexcitation of glioma cells is theorized to facilitate tumor invasion, and direct in vivo optogenetic excitation of glioma cells has been shown to promote tumor proliferation [19–21]. While this finding appears contradictory to an association between seizure presence and improved prognosis in HGG, at least one study has demonstrated a possible association between the anti-seizure medication (ASM) lamotrigine and delayed tumor progression in optic pathway glioma [22]. We hypothesize that the suppressive effects of ASM use on neuronal excitability may underpin the prognostic benefit of a positive seizure history in HGG.

Several ASMs have also shown a cytotoxic effect towards glioblastoma cells in vitro. One clinical study of 249 patients with tumor-related epilepsy demonstrated better survival in patients receiving ASM therapy compared to patients not taking ASMs, though it is challenging to discern survival related to seizure control versus antiseizure medication effect [23, 24]. Further, we report an increase in overall and progression-free survival in patients who achieved partial or complete seizure control compared to patients who were unable to achieve six seizure-free months. The 2024 Pallud et al. retrospective analysis reported less epileptic control at the time of tumor progression; our study elaborates further by demonstrating a significant association between seizure control and both improved PFS and OS [15]. Additional studies, including comparison of outcomes between HGG patients receiving different ASMs, are needed to validate the hypothesis that ASMs reduce HGG tumor progression and understand if ASM therapy could be better leveraged to improve HGG outcomes.

When analyzing only known *IDH1*-wildtype patients, the significant associations observed in our full 950 patient analyses were lost. However, the directionality and approximate magnitude of hazard ratios in the 563-patient *IDH1*-wildtype analyses mimicked those seen in the 950-patient analyses, a finding we propose may speak to a loss in statistical power with reduced sample size [25]. Further, a 2024 retrospective analysis looking exclusively at *IDH*-wildtype cases also demonstrated prolonged survival with seizure presence at diagnosis [15]. Alternatively, these findings may be influenced by changing treatment practices over time– *IDH1* testing primarily occurred after 2015. When plotting anticancer treatments received over time, no obvious temporal shifts are seen, although more subtle variation in specific medications or dosing could be possible (Supplementary Fig. 3).

A limitation of this study is its retrospective nature, which allows for the introduction of recall bias. While we extracted data from a prospectively collected clinical database for the study of CNS tumors, all variables related to tumor-related epilepsy were collected retrospectively. For example, EEG collection is not part of the standard of care for HGG-associated epilepsy in most cases; we relied on clinical documentation of seizure activity. Our large sample size, however, does help to reduce this bias. Additionally, our study was conducted without access to tumor volume data; determining whether seizure activity correlates with tumor volume would be a prudent future research direction to clarify whether patients with seizures are diagnosed with smaller tumors.

Our investigation should serve as retrospective evidence that the positive prognostic value of epileptic activity in patients with HGG goes beyond the early-diagnosis theory. Future exploration of molecular alterations in HGG to assess their potential impact on seizure incidence and management, as well as disease course, is warranted. Despite the potential positive prognostic value of epileptic activity in HGG survival, seizures nonetheless can have a serious detrimental impact on quality of life, and our findings demonstrate a need for greater understanding of late-onset epileptic activity and better approaches to management. Seizure presence associates with improved overall survival, but seizure control is affected by many factors including the selection of ASMs, and additional study is needed to understand how the clinical approach to treating HGG-associated epilepsy affects HGG disease course and survival.

## Electronic supplementary material

Below is the link to the electronic supplementary material.

Supplementary Material 1

## Data Availability

No datasets were generated or analysed during the current study.

## Figures

Study figures were generated using R.
