# Supplementary material for: Tumor-related epilepsy in high-grade glioma: a large series survival analysis
Source: J Neurooncol. 2024 Aug 5;170(1):153–60. doi: 10.1007/s11060-024-04787-z (PMC11447087; doi:10.1007/s11060-024-04787-z)
Supplement: Supplementary file 1 — Supplementary Material 1 [file 11060_2024_4787_MOESM1_ESM.pdf]

Tumor-related epilepsy in high-grade glioma: a large series survival analysis

Ryan G. Rilinger, BA[1,2]; Lydia Guo, BA[1,2]; Akshay Sharma, MD[2]; Josephine Volovetz, MD[2]; Nicolas R. Thompson, MS[3,4]; Matthew Grabowski, MD[2]; Mina Lobbous, MD[1,5]; Andrew Dhawan, MD, DPhil[1,6]

[1]Cleveland Clinic Lerner College of Medicine, [2]Department of Neurosurgery, Cleveland Clinic Foundation, [3]Lerner Research Institute Quantitative Health Sciences Department, [4]Neurological Institute Center for Outcomes Research & Evaluation [5]Department of Neuro-Oncology, Cleveland Clinic Foundation, [6]Rose Ella Burkhardt Brain Tumor and Neuro-Oncology Center

Corresponding author: Andrew Dhawan, MD
Email: dhawana@ccf.org
Phone: 216-445-2428
Address: 9500 Euclid Avenue, Cleveland OH 44195
ORCID: 0000-0002-5027-1277

Supplementary Materials

Supplementary Table 1. Additional patient and clinical characteristics, stratified by seizure status. "Statistic" is "No. (%)" unless noted otherwise.

	All Patients		No Seizures		Early Seizures (pre-HGG diagnosis		Late Seizures (post-HGG diagnosis)		P-value
	**N**	**Statistic**	**N**	**Statistic**	**N**	**Statistic**	**N**	**Statistic**	
**Tumor Laterality**									
Left	949	441 (46.5%)	536	246 (45.9%)	260	124 (47.7%)	153	71 (46.4%)	0.093
Right		461 (48.6%)		255 (47.6%)		131 (50.4%)		49.0%)	
Bilateral		47 (5.0%)		35 (6.5%)		5 (1.9%)		7 (4.6%)	
**Tumor Location**									
Frontal Lobe	950	340 (35.8%)	536	166 (31.0%)	261	107 (41.0%)	153	67 (43.8%)	0.002
Parietal Lobe	950	281 (29.6%)	536	158 (29.5%)	261	65 (24.9%)	153	58 (37.9%)	0.020
Temporal Lobe	950	356 (37.5%)	536	207 (38.6%)	261	111 (42.5%)	153	38 (24.8%)	0.001
Occipital Lobe	950	100 (10.5%)	536	70 (13.1%)	261	17 (6.5%)	153	13 (8.5%)	0.012
Other Location	949	130 (13.7%)	536	95 (17.7%)	260	18 (6.9%)	153	17 (11.1%)	< 0.001
**KPS at Diagnosis, median (IQR)**	898	80 (70, 90)	494	80 (70, 90)	257	90 (80, 90)	147	80 (80, 90)	< 0.001
**IDH1 status available**	600	37 (6.2%)	339	14 (4.1%)	150	19 (12.7%)	111	4 (3.6%)	< 0.001
**Initial Seizure Frequency**									
Median (IQR)	407	1 (0, 1)	NA	NA	255	1 (1, 2)	152	0 (0, 0)	< 0.001
0	407	149 (36.6%)	NA	NA	255	18 (7.1%)	152	131 (86.2%)	< 0.001
1		165 (40.5%)	NA	NA		154 (60.4%)		11 (7.2%)	
2+		93 (22.9%)	NA	NA		83 (32.5%)		10 (6.6%)	

	All Patients		No Seizures		Early Seizures (pre-HGG diagnosis		Late Seizures (post-HGG diagnosis)		P-value
	N	Statistic	N	Statistic	N	Statistic	N	Statistic	
**ASMs Received**									
Gabapentin	422	21	NA	NA	274	16	148	5	< 0.001
Lacosamide		65		NA		40		25	
Lamotrigine		37		NA		26		11	
Levetiracetam		371		NA		240		131	
Perampanel		13		NA		10		3	
Phenytoin		72		NA		56		16	
Topiramate		12		NA		5		7	
Valproic acid		13		NA		8		5	
Zonisamide		36		NA		23		13	
Other		30		NA		24		6	

Supplementary Table 2. Classification of chemotherapy. Drugs are listed in order of most commonly to least commonly prescribed; number of patients receiving each drug is represented in parentheses. Generic names are used except for investigational products without an applicable generic name yet.

**Cytotoxic chemotherapy**	
**Drug class**	**List of drugs**
Alkylating agent	temozolomide (761), lomustine (177), carboplatin (34), carmustine (15), dianhydrogalactitol (4), procarbazine (3)
Calcineurin inhibitor	cyclosporine (27)
Retinoids	cis-retinoic acid (38)
Topoisomerase inhibitor	etoposide (83), irinotecan (46), topotecan (5), govitecan (2)
Uncommon/other	capecitabine (7), vorinostat (7), VT1021 (7), hydroxyurea (7), perillyl alcohol (6), everolimus (3), thalidomide (3), AT-101 (gossypol enantiomer) (4), flucytosine (2), hydroxychloroquine (2), tamoxifen (2), vincristine (2), vocimagene amiretrorepvec (2), anastrozole (1), eflornithine (1), enzasturin (1), ixabepilone (1), lucanthone (2), methotrexate (1), paclitaxel trevatide (1), sirolimus (1), terameprocol (1), CBL0137 (a curaxin) (1), D2C7-IT (1)

**Targeted chemotherapy**	
**Drug class**	**List of drugs**
Kinase inhibitor	ruxolitinib (33), erlotinib (28), ibrutinib (17), dovitinib (7), regorafenib (7), dacomitinib (5), tandutinib (5), cediranib (4), neratinib (3), osimertinib (3), paxalisib (3), trametinib (3), dabrafenib (2), galunisertib (2), imatinib (2), lapatinib (2), sorafenib (2), abemaciclib (1), adavosertib (1), erdafitinib (1), pemigatinib (1), sapanisertib (1), sunitinib (1), tesavatinib (1), voxtalisib (1)
Monoclonal antibody	bevacizumab (327)
Uncommon/other inhibitors	RO4929097 (6), cilengitide (5), methoxyamine (4), ONC201 (3), selinexor (2), veliparib (2), bortezomib (1), iniparib (1), ivosidenib (1), ONC206 (1), PT2385 (2), veledimex (1), vismodegib (1), unidentified PARP-1 inhibitor (3)
**Immunotherapy**	
**Drug class**	**List of drugs**
Monoclonal antibody	nivolumab (33), pembrolizumab (30), carotuximab (4), olaratumab (4), sacituzumab (2), anti-LAG-3 (1), panitumumab (1), 2141-V11 (1)
Vaccine	SVN53-67/M57-KLH (SurVaxM) (28), rindopepimut (7), PEP-CMV vaccine (1), rWTC-MBTA (DCVax-L) (1), ICT-107 (dendritic cell vaccine) (1)
Uncommon/Other	IL-13 (8), poly-ICLC (6)

Supplementary Figure 1: Anticancer therapy received over time. Anticancer therapy is categorized as radiation therapy, or one of three medication classes as defined in Supplementary Table 2.

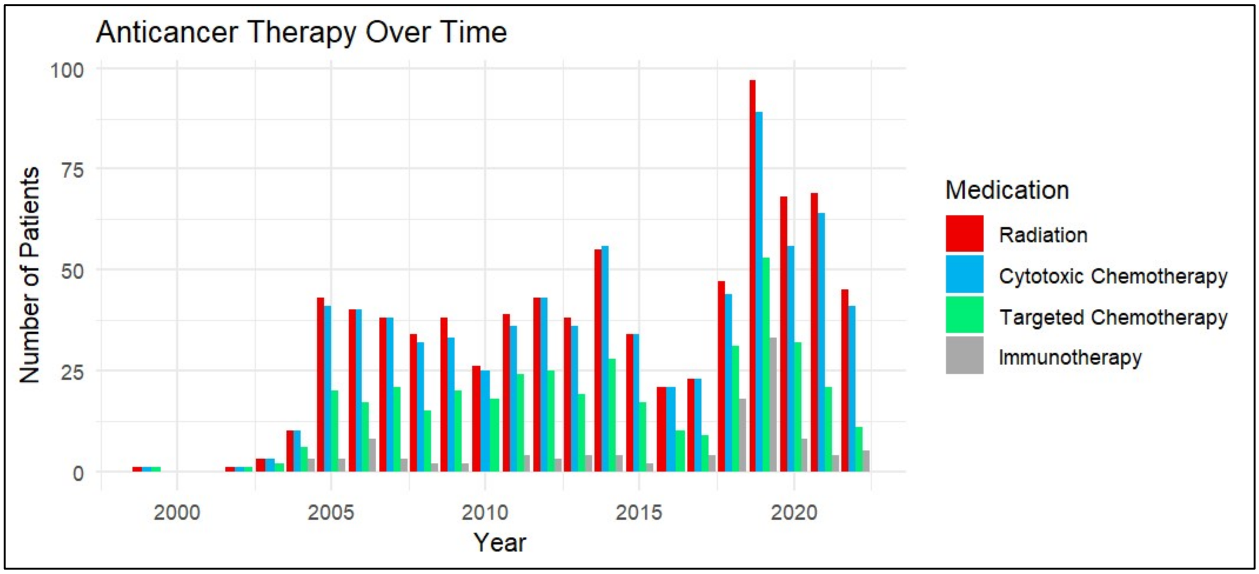

Supplementary Table 3. Multivariable Cox proportional hazard models for overall survival and progression-free survival. Model restricted to only include patients who received anticancer therapy (radiation, cytotoxic chemotherapy, and/or targeted chemotherapy). Separate models were fit for each outcome and independent variable: seizure timing (Early and Late seizure vs. No seizures), frequency (number of seizures at initial HGG presentation), and control (Partial or Complete control vs. No control).

		Hazard Ratio (95% CI)	P-value	Omnibus P-value[b]
**Overall Survival Models (All patients; N=829)**				
Timing (vs. No seizure)	Early seizure	0.82 (0.68, 0.97)	0.025	0.016
	Late seizure	0.77 (0.62, 0.95)	0.015	
	Any seizure	0.80 (0.68, 0.93)	0.005	NA
Restricted Timing (vs. No seizure)[a]	Early seizure	0.81 (0.68, 0.97)	0.024	0.072
	Late seizure	0.96 (0.76, 1.20)	0.711	
	Any seizure	0.86 (0.73, 1.01)	0.058	NA
Frequency (vs. 0)	1	1.00 (0.78, 1.29)	0.983	0.607
	2+	1.15 (0.85, 1.55)	0.359	
Seizure control (vs. No control)	Partial control	0.29 (0.21, 0.42)	< 0.001	< 0.001
	Complete control	0.41 (0.32, 0.53)	< 0.001	
**Progression-Free Survival Models (All patients, N=829)**				
Timing (vs. No seizure)	Early seizure	0.90 (0.76, 1.07)	0.248	0.253
	Late seizure	0.86 (0.70, 1.05)	0.132	
	Any seizure	0.89 (0.76, 1.03)	0.114	NA
Restricted Timing (vs. No seizure)[a]	Early seizure	0.91 (0.77, 1.08)	0.270	0.335
	Late seizure	1.07 (0.86, 1.33)	0.551	
	Any seizure	0.96 (0.82, 1.11)	0.558	NA
Frequency (vs. 0)	1	0.92 (0.72, 1.17)	0.502	0.289
	2+	1.16 (0.86, 1.55)	0.332	
Seizure control (vs. No control)	Partial control	0.46 (0.33, 0.65)	< 0.001	< 0.001
	Complete control	0.56 (0.44, 0.72)	< 0.001	
**Overall Survival Models (*IDH1* status known, N=479)**				
Timing (vs. No seizure)	Early seizure	0.85 (0.67, 1.10)	0.215	0.333
	Late seizure	0.85 (0.64, 1.11)	0.230	
	Any seizure	0.85 (0.69, 1.05)	0.138	NA
Restricted Timing (vs. No seizure)[a]	Early seizure	0.85 (0.66, 1.09)	0.203	0.436
	Late seizure	0.92 (0.69, 1.23)	0.577	
	Any seizure	0.88 (0.71, 1.09)	0.233	NA
Frequency (vs. 0)	1	0.96 (0.68, 1.36)	0.820	0.094
	2+	1.54 (1.00, 2.37)	0.049	

		Hazard Ratio (95% CI), continued	P-value, continued	Omnibus P-value, continued
**Progression-Free Survival Models (*IDH1* status known, N=479)**				
Timing (vs. No seizure)	Early seizure	1.03 (0.81, 1.30)	0.822	0.470
	Late seizure	0.87 (0.68, 1.13)	0.297	
	Any seizure	0.96 (0.78, 1.17)	0.656	NA
Restricted Timing (vs. No seizure)[a]	Early seizure	1.03 (0.81, 1.30)	0.826	0.941
	Late seizure	0.97 (0.74, 1.28)	0.852	
	Any seizure	1.01 (0.82, 1.23)	0.954	NA
Frequency (vs. 0)	1	1.05 (0.76, 1.45)	0.788	0.093

[a] "Restricted Timing" = model including only Late Seizure patients with seizure onset within 14 months of HGG diagnosis.

[b] Omnibus test is not applicable for the Any seizure vs. No seizure models.
